# Longitudinal multiphoton imaging of cerebral amyloid angiopathy in response to anti-ApoE4 immunotherapy in mice

**DOI:** 10.1186/s13024-026-00957-x

**Published:** 2026-06-11

**Authors:** Orla Bonnar, Fareeha Saadi, Maria V. Sanchez-Mico, Lee H. Hanlin, Kali A. Vom Eigen, Nicole Mumbi, Brian J. Bacskai, Steven M. Greenberg, David M. Holtzman, Susanne J. van Veluw

**Affiliations:** 1 MassGeneral Institute for Neurodegenerative Disease, Massachusetts General Hospital and Harvard Medical School, Charlestown Navy Yard, Charlestown, MA 02129 USA; 2 Department of Neurology, Hope Center for Neurological Disorders, Charles F. and Joanne Knight Alzheimer’s Disease Research Center, Washington University School of Medicine, St. Louis, MO 63110 USA; 3 J. Philip Kistler Stroke Research Center, Department of Neurology, Massachusetts General Hospital and Harvard Medical School, Boston, MA 02114 USA; 4 UK Dementia Research Institute – Centre for Vascular Dementia Research, University of Edinburgh, Edinburgh, EH16 4SB UK

**Keywords:** APOE, Apolipoprotein, Cerebral amyloid angiopathy, Alzheimer’s disease, ARIA

## Abstract

**Background:**

There are no available treatments to halt or slow the progression of cerebral amyloid angiopathy (CAA), a disease neuropathologically characterized by the deposition of amyloid-β (Aβ) within the walls of the cerebrovasculature. Recently a novel therapeutic strategy has been described, targeting non-lipidated ApoE4 that co-deposits with Aβ, resulting in lower levels of Aβ across the brain. To understand the therapeutic potential for patients with CAA, we sought to determine if this global reduction in Aβ deposits corresponds to the active removal of existing aggregates in the vasculature and if so, whether this may improve vascular function over time.

**Methods:**

Cranial windows were implanted in 9-10-month-old 5xFAD mice expressing human APOE4 to facilitate chronic, unanesthetized imaging using in vivo multiphoton microscopy. Mice were treated weekly with anti-ApoE4 immunotherapy (HAE-4) or control IgG (50 mg/kg). Parenchymal and vascular Aβ burden as well as vascular function were measured in vivo before and during treatment. Post-mortem brains were assessed for CAA, parenchymal Aβ plaques and iron deposits. In a separate study, 5xFAD mice were treated with weekly HAE-4 or control IgG with the same doses of antibodies from 8 to 10 months of age in the absence of cranial windows.

**Results:**

Treatment with HAE-4 resulted in reduction of total Aβ plaque area post-mortem in mice and shrinkage of existing smaller plaques imaged with in vivo multiphoton microscopy. Vascular fibrillar Aβ under the cranial window conversely increased over time either with or without HAE-4 treatment and there was no treatment-associated improvement in vascular function in cortical arterioles in the areas measured in vivo. There was no evidence of hemorrhagic events linked to treatment, however there was significant immune cell activation. In 5xFAD mice treated without a cranial window, there was a reduction in plaques and CAA as previously described in HAE-4 vs. control treated mice.

**Conclusions:**

Anti-ApoE4 immunotherapy, as shown previously, decreased the overall amount of Aβ. It also appeared to remove some existing plaque Aβ without measurable effects on vascular fibrillar Aβ deposits or vascular function in areas measured in vivo under a cranial window. The absence of treatment-associated hemorrhagic events may offer a comparative advantage relative to anti-Aβ immunotherapy.

**Supplementary Information:**

The online version contains supplementary material available at 10.1186/s13024-026-00957-x.

## Introduction

Cerebral amyloid angiopathy (CAA) is a cerebrovascular disease that is associated with cognitive decline and is a strong risk factor for intracerebral hemorrhage [1]. It is neuropathologically characterized by the deposition of amyloid-β (Aβ) within the walls of leptomeningeal and cortical arterioles [1]. CAA exhibits large pathological overlap with Alzheimer’s disease (AD) where Aβ deposits as parenchymal plaques, and between 50 and 80% of patients with AD also have CAA [2]. Despite its devastating clinical phenotype and high prevalence among the elderly, there are currently no disease-modifying treatment options available for patients with CAA. Recently approved anti-Aβ immunotherapies are associated with Amyloid-Related Imaging Abnormalities (ARIA) which present as either ARIA-E (edema) or ARIA-H (hemorrhage) [3, 4]. This risk is further elevated in individuals with CAA and so anti-Aβ immunotherapy with currently available FDA approved antibodies, is not a recommended treatment option for this patient group [5].

Possession of the ε4 allele of the apolipoprotein E (APOE) gene increases the risk of developing CAA, is associated with a greater deposition of vascular Aβ, and increases the risk of developing ARIA with administration of anti-Aβ immunotherapy [6–9]. This heightened risk profile makes treating APOE4 carriers with CAA particularly challenging and represents an urgent clinical need.

Recent work in a 5XFAD mouse model crossed with mice expressing human APOE4 showed that promise lies with anti-ApoE4 immunotherapy (HAE-4) as an alternative to anti-Aβ immunotherapy [10]. Specifically, non-lipidated ApoE4 that is co-deposited with parenchymal Aβ plaques and CAA was targeted [11], and mice that were treated for eight weeks had less Aβ present along the vasculature and in the brain’s parenchyma. Critically, there was no evidence for concomitant hemorrhagic events in HAE-4 treated animals, unlike those treated with aducanumab. Further, acutely measured vascular reactivity, measured just prior to sacrifice, was superior in mice that received immunotherapy compared to those who received a control antibody [10]. This study raised several questions that are critical to our understanding of how this treatment might be used as a therapeutic in CAA. Namely, can HAE-4 immunotherapy actively remove existing vascular Aβ and if so, does this alter vascular function of these individual vessels over time. To answer these questions, we utilized the 5XFAD APOE4 mouse model and administered HAE-4 or control IgG (50 mg/kg) weekly for 12 weeks, starting at 10 months of age. We used multiphoton microscopy to longitudinally observe the effects of treatment on existing CAA and parenchymal Aβ plaque deposits across the same fields of view (FOV), as well as to measure any alterations in vascular function over time. In line with previous work [10], we saw that the brains of mice who received HAE-4 immunotherapy had fewer Aβ deposits post-mortem after the completion of treatment, without an increase in hemorrhages. This reduction in HAE-4 treated mice was associated with an increase in activated microglia around CAA positive vessels. We did not observe any removal of existing CAA with in vivo multiphoton microscopy but did see a shrinkage of the smallest parenchymal Aβ plaques. Further, we did not see an alteration of vascular function with treatment under the window, in accordance with the observed unchanging vascular Aβ burden. Together, these results suggest that HAE-4 treatment could be an effective and safe treatment strategy for the removal of small Aβ plaques, without an associated risk of hemorrhage. However, it does not appear to successfully remove existing fibrillar CAA from the vasculature in areas measured in vivo. It appears to prevent the aggregation of new plaques and new CAA deposits as shown previously, though future studies that include vessels without existing vascular Aβ deposits at baseline, prior to treatment initiation, should be carried out to confirm this.

## Materials and methods

### Animals

All animal procedures were performed with the approval of the Massachusetts General Hospital (MGH) Animal Care and Use Committee and in compliance with the National Institutes of Health Guide for the Care and Use of Laboratory Animals. Nine- to ten-month-old male and female 5XFAD^+/+^/APOE4^+/+^ and 5XFAD^−/−^/APOE4^+/+^ littermates were used throughout the study. Mice were generated by crossing 5xFAD mice (line Tg7031) with human APOE4 knock-in mice (apoE4^+/+^) [12] at the Washington University School of Medicine (henceforth referred to as 5xE4 mice). Male 5XFAD^+/+^/APOE4^+/+^ were bred to female APOE4^+/+^ to produce all animals in this study as described [13]. Mice were housed singly following surgery at MGH with ad libitum access to food and water on a 12-hour light dark cycle.

### Antibody generation

HAE-4 and control IgG are IgG2ab isotype and were generated as described previously [11]. The antibodies were produced at NextCure. Antibodies were produced from stably-transduced CHO cell cultures and purified to ≥ 97% purity using Protein A chromatography. Each eluted antibody was formulated in a buffer of 20 mM Histidine, 8.7% (w/w) sucrose, and 0.02% (w/w) Polysorbate-80 at pH 5.5. Antibodies were stored at -80 °C until administration.

### Surgical procedures

Chronic cranial windows were implanted over the right visual cortex as described previously [14]. In brief, animals were anesthetized using isoflurane 5% at induction and maintained at 1-2.5%. Animals were placed in a custom stereotaxic frame and secured using ear bars. Hair was removed from the scalp using commercially available hair removal cream and scalp was removed exposing skull beneath. To improve adhesion, the skull was scratched using forceps and any remaining membrane was removed. A 3 mm craniotomy was created over the primary visual cortex and was plugged with a stacked coverslip held in place and secured using dental cement mixed with KrazyGlue. Custom made stainless steel headposts were attached using C & B metabond (Parkell) to facilitate head fixation. Meloxicam (5 mg/kg) and acetaminophen (300 mg/ml) were provided as analgesia.

### Treatment paradigm

**Fig. 1:**
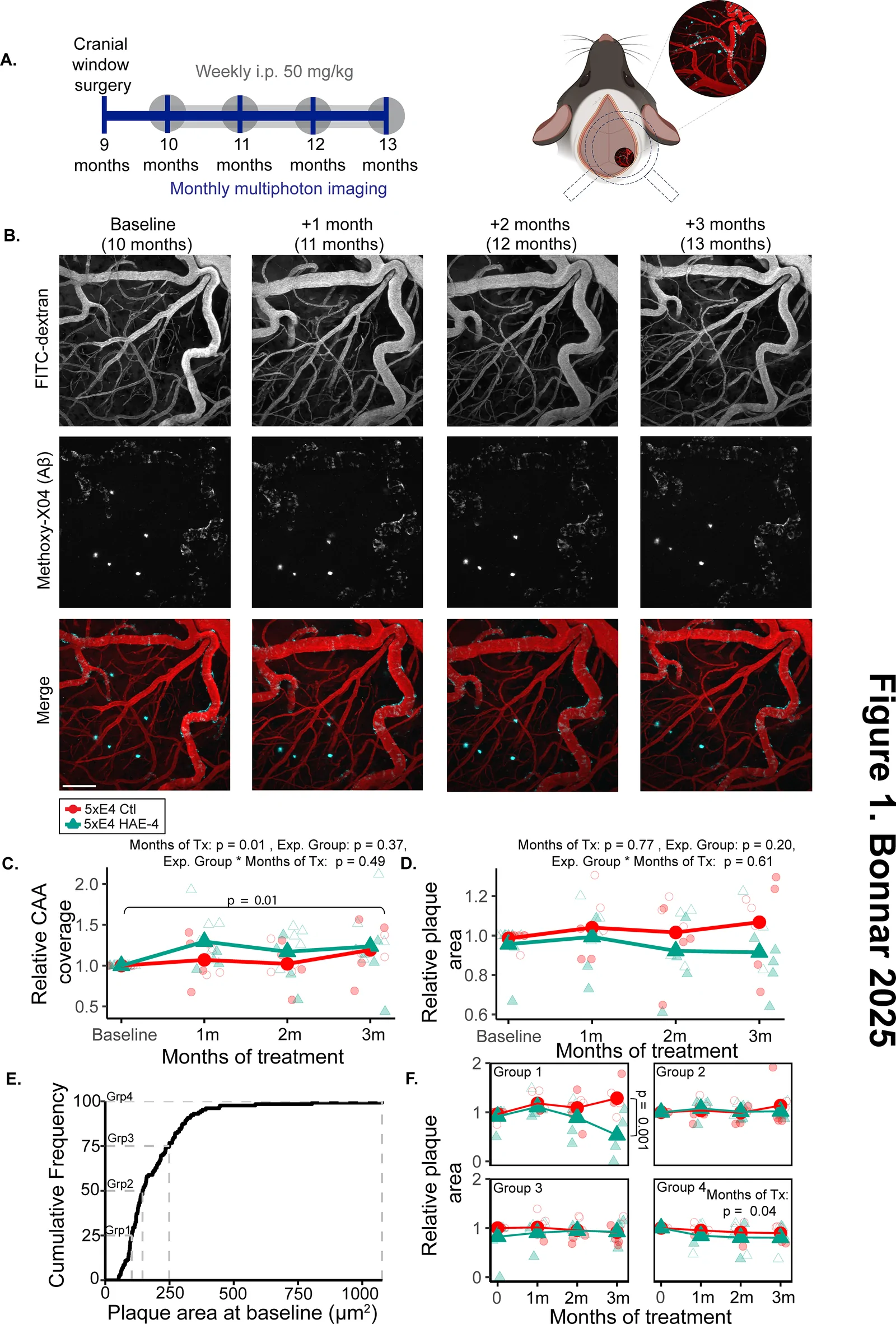
HAE-4 treatment does not remove existing CAA but causes a shrinkage of the smallest plaques. (**A**) Experimental paradigm. 5XFAD x APOE4 (5xE4) mice were equipped with a chronic cranial window at 9 months (right panel; Created in BioRender. https://BioRender.com/eh7wel4) and one month later underwent a baseline imaging session before the initiation of treatment. Mice then received weekly (50 mg/kg) intraperitoneal injections of HAE-4 or control IgG for 3 months. (**B**) The same field of view was imaged before the initiation of treatment and monthly throughout the treatment duration. The vascular lumen was labelled with FITC-dextran (pseudocolor red) and Aβ deposits were labelled with methoxy-X04 (cyan). Scale bar = 100 μm. (**C**) CAA burden increased with age (1 month vs. 3 months, *p* = 0.01) but there was no effect of treatment. (**D**) There was no significant effect of treatment on total parenchymal plaque area. When plaques were split into equally sized groups based on area (**E**) there was a significant reduction of the smallest plaques (group 1, [*p* = 0.001]) in mice treated with HAE-4. There was also an effect of imaging session (*p* = 0.04) in the largest plaques (group 4). No effect of sex was observed in C or D (F lacked statistical power to test for sex). Reported p-values from two-way repeated ANOVAs/mixed-effects analysis. Each data point represents the animal average and solid lines (in **C, D**, and **F**) represent average per treatment group. Males are represented by open symbols and females are represented by filled symbols. *N* = 7–10 animals per group (4–5 females / 3–4 males per group)

5xE4 animals were treated weekly with either 50 mg/kg of HAE-4 anti-ApoE antibody or control IgG for 12 weeks, administered intraperitoneally. The experimenter was blinded to experimental group during dose preparation, injections, data collection, and analysis, and imaging fields of view were selected before the initiation of treatment. Littermates that were negative for 5XFAD were used as controls and were administered PBS for 12 weeks. The absence of Aβ in the control animals precluded blinding in this part of the study. Animals were imaged (as described below) at baseline (before the administration of HAE-4, control IgG, or PBS), and monthly during 12 weeks of treatment with the final session following the last dose of antibody. Within one week of the final dose, mice were sacrificed, and brains were extracted for post-mortem study as described in further detail below. See Fig. 1A for an overview. Not all mice could be imaged at all four time points due to window deterioration as displayed in data figures.

### Two-photon microscopy

Mice were imaged on a FluoView FV1000MPE system (Olympus) mounted on a BX61WI microscope with a 25x (NA: 1.05) water immersion objective (Olympus). A Spectra Physics MaiTai HP or Insight X3 laser was used to stimulate at 800 nm, and external photomultiplier tubes (PMTs, Hamamatsu) were used to detect light. In order to maintain consistency between consecutive imaging sessions, PMTs were kept consistent per mouse, however laser power was occasionally adjusted if necessary, so that there were no saturated pixels in CAA when using a HiLo look-up-table. In the week prior to imaging, mice were habituated to the awake imaging set up. The day prior to imaging, mice were injected intraperitoneally with methoxy-X04 (~ 5 mg/kg), dissolved in 3% Cremophor and PBS to allow for the visualization of Aβ. Immediately prior to imaging, animals were anesthetized briefly using isoflurane (3–5%) and were injected retro-orbitally with 200 µL Fluorescein Isothiocyanate (FITC)-Dextran (70,000 mW, Invitrogen, 12.5 mg/ml) to label plasma. Animals were then headfixed on a circular treadmill where they were free to walk but the skull remained in place to facilitate imaging through the cranial window. During visual stimulation experiments a flashing checkerboard (8 Hz) was presented to the left eye for 2 s with an 8 s inter-stimulus interval (resulting in a low frequency stimulation of 0.1 Hz). All other images were acquired under identical conditions with the mouse in the dark.

### Vascular and CAA imaging

Between one and four cortical volumes were taken per mouse to measure Aβ plaque and CAA burden over time (1x magnification, pixel size 0.8 μm, 4 µs/pixel, 640 × 640 pixels in size, 1 μm z-step size). Between one and two pial arterioles were identified within separate FOVs and vascular diameter time series were recorded both with and without visual stimulation (2x magnification, pixel size 0.99 μm, 2 µs/pixel, 256 × 256 pixels in size, 700 frames [5 min]). Arteriolar diameter changes due to cardiac pulsations were recorded using high speed line scans (10,000 lines, 2.5 s).

### In vivo analysis

Percentage vessel coverage with CAA and parenchymal plaque area was measured in the same FOV across all imaging sessions. Both the methoxy-X04 channel and vessel channel were auto-thresholded in Fiji: ImageJ [15]. In some cases, the threshold was adjusted manually as needed. To quantify CAA using custom ImageJ / MATLAB scripts (R2022b, MathWorks), a vascular ROI was drawn and expanded by 5 pixels. The area of methoxy-X04 was normalized to the vascular area to yield a percentage area covered. Similarly, plaque area (methoxy-X04) was measured in each FOV. This was repeated during each imaging session with care taken to measure the same vessel segment and same plaques. For visual stimulation experiments, data were cut into 10 s trials matching the visual stimulation paradigm and normalized to the first frame of the trial. All trials were averaged per vessel and area under the curve (AUC) was determined using the inbuilt MATLAB function ‘trapz’ which utilizes trapezoidal numerical integration. To measure low frequency vascular diameter oscillations, a fast Fourier transform was conducted and the AUC of a low frequency range (0.05–0.15 Hz) was determined. To measure vascular beat-to-beat pulsatility, the integral of the diameter trace generated from high-speed line scans was determined. All data were normalized to the first imaging session and averaged per mouse so that relative change is reported throughout the manuscript.

### Ex vivo tissue processing and analysis

Mice were transcardially perfused with ice-cold PBS and brains were immediately extracted. The non-surgical hemisphere was fixed in 4% paraformaldehyde and 15% glycerol solution and was later processed, embedded in paraffin, and cut into 6 µm thick sections. Slides were stained for Perls’ Prussian blue to detect iron deposits by incubation in 1:1 concentration of 5% hydrochloric acid and 5% Potassium Ferrocyanide for 30 minutes and were counterstained with filtered neutral red for 1 minute. Immunohistochemistry was performed against Aβ (clone 6F/3D, Agilent, 1:200, Cat# M0872) using a standardized protocol. Briefly, samples were dewaxed and rehydrated using a xylene and ethanol gradient, endogenous peroxidases were inhibited by incubating in 3% H_2_O_2_ for 30 minutes followed by antigen retrieval in heated citrate buffer (pH 6) for 20 minutes. Samples were blocked for 30 minutes using normal goat or horse serum (Vectastain ABC kit, Vector Laboratories) and were then incubated with the primary antibody overnight at 4°C. The next morning, sections were brought to room temperature and washed with PBS, followed by an incubation with the biotinylated secondary antibody for 1 hour (Vectastain ABC kit, Vector Laboratories). Slides were then incubated with an avidin-biotin solution (Vectastain ABC kit, Vector Laboratories) and finally were developed for 3–5 minutes with 3,3’-diaminobenzidine (DAB, Vector Laboratories). Finally, sections were co-stained with hematoxylin, dehydrated using an ethanol gradient, and cleared in xylene. Slides were immediately cover slipped using Permount mounting medium.

Slides were scanned using a NanoZoomer Digital Pathology whole slide scanner (C9600-12 Hamamatsu Photonics KK, Japan) with a 40x objective (221 nm/pixel). Image analysis took place in QuPath [16] where a random trees pixel classifier (high resolution: 0.88 μm/pixel) was trained on a subset of images. With the user blinded to the DAB channel, regions of interest (ROIs) were manually selected over the cortex of each imaged section and the pixel classifier was applied. Classified images were manually inspected for quality control and any classified pixels that were outside of the tissue area were deleted. Upon inspection of images, sections from two mice were excluded due to high background signal, likely due to a poor perfusion. Subsequently, all pixels classified as Aβ were manually classified as ‘CAA’ or ‘plaque’. For sections stained with Perls’ Prussian blue, iron-positive ROIs were manually drawn and a trained random trees pixel classifier (high resolution: 0.88 μm/pixel) was then used to quantify the area of iron positive pixels within the ROIs. This was normalized to the tissue area as measured by a trained random trees pixel classifier (moderate resolution: 1.76 μm/pixel). For Aβ quantification, 2–4 sections contributed to the animal average and for Prussian blue staining, 8 sections were averaged per animal.

### Statistics

Statistical analysis was conducted in GraphPad Prism 9 and Rstudio (R version 4.2.2 [2022-10-31]) [17]. All data were tested for normality using a Shapiro-Wilk test. If all experimental groups were normally distributed, data were analyzed with a student’s t-test or two-way repeated ANOVA/mixed-effects analysis. Mann-Whitney U or Kruskal Wallis tests were used when all groups were not normally distributed. When necessary, post-hoc comparisons were made with a Bonferroni’s correction. For correlation analyses, data were not normally distributed and so a Spearman’s ρ test was conducted. In all datasets with sufficient power to test for the effect of sex (only HAE-4 or control IgG as PBS treated group was not well balanced for sex), we included this in the analysis. In longitudinal experiments, the effect of sex was examined in the final imaging session only. Across all measures there was no observed effect of sex and so this was not reported in primary analysis outputs.

## Results

### Treatment with HAE-4 reduced parenchymal plaque size, but did not remove existing CAA

One of the aims of this study was to determine whether HAE-4 immunotherapy can actively clear deposited CAA from the vasculature. The CAA and Aβ plaques were identified in vivo using methoxy-X04 and thus only identified Aβ fibrils. 5XFAD mice were crossed with mice expressing human APOE4 (5xE4 mice) to longitudinally measure CAA coverage and parenchymal Aβ fibril deposition at the level of individual vessels and plaques over time. These mice begin to develop Aβ pathology at around 6 months of age and so we elected to begin treatment at 10 months at which time there is a moderate level of vascular Aβ fibrils, therefore more closely recapitulating what might be observed in patients with early CAA. We used multiphoton microscopy to chronically and concomitantly visualize the vasculature and Aβ fibrils in awake animals. Before treatment began, mice underwent a baseline imaging session where pial vessels with moderate CAA deposition were identified and recorded. Mice were then administered HAE-4 or control IgG (50 mg/kg) weekly for 12 weeks and underwent monthly imaging sessions throughout the treatment duration **(**Fig. 1A-B**)**. This dose was selected based on previous work demonstrating that the half-life of HAE-4 injected peripherally is approximately 7 days and 50 mg/kg demonstrated efficacy in reducing Aβ burden [11]. To determine if any existing Aβ fibrils were removed as a result of HAE-4 treatment, the percentage CAA coverage of the same vessels, and the area of the same individual plaques was recorded at each imaging session for the duration of treatment **(**Fig. 1B**)**. We did not observe any removal of CAA from the pial vessels that started with moderate Aβ burden. In fact, we saw an increase in CAA coverage after 3 months in both treatment groups **(**Fig. 1C**)**. We did not see a significant decrease in the total area of parenchymal Aβ fibrillar plaques **(**Fig. 1D**)**, however, we hypothesized that smaller plaques would be easier to clear than larger, denser plaques. Accordingly, we split data into four equally sized groups according to plaque size **(**Fig. 1E**)**, ranging from the smallest to the largest. We observed a significant shrinkage of the smallest plaques (group 1) in mice treated with HAE-4, but not in larger plaques (groups 2–4) **(**Fig. 1F**)**. It should be noted that there was also an overall effect of imaging session in the largest plaques (group 4, *p* = 0.04), but no effect of treatment group. When interrogated further, we found that this was driven by a trend in the reduction of plaque size in HAE-4 treated mice between baseline and 3 months of treatment (*p* = 0.07). Given that no significant effect of treatment group was present, further experiments would be required to unravel the effects of HAE-4 treatment on larger plaques.

Together these data suggest that there was a modest shrinkage of the smallest parenchymal fibrillar plaques but that fibrillar CAA on pial arterioles was not actively removed by HAE-4 in regions of the brain where in vivo imaging occurred.

### Treatment with HAE-4 did not alter spontaneous vascular function of pial vessels over time

**Fig. 2:**
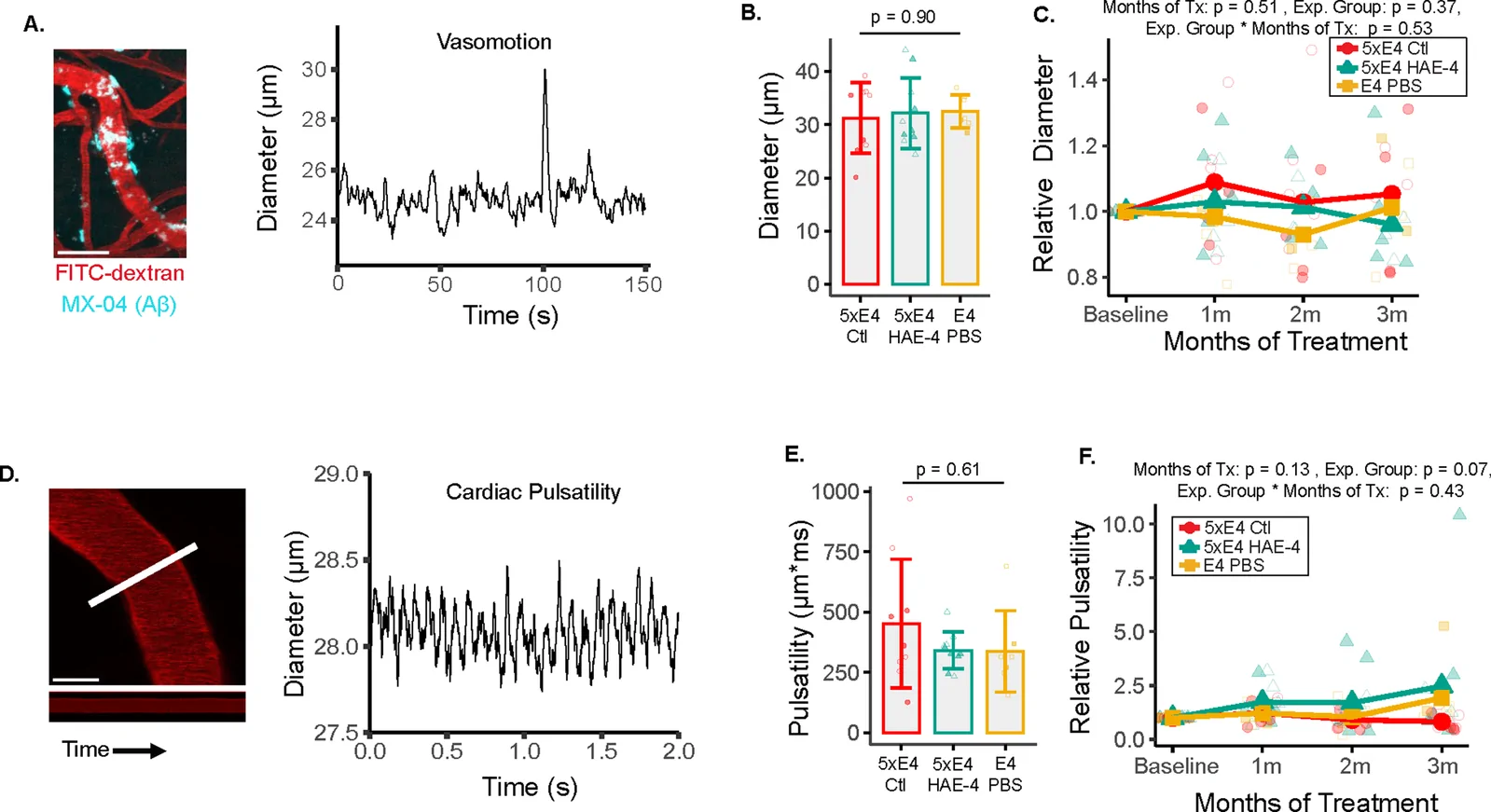
HAE-4 treatment does not affect pial vessel diameter or beat-to-beat cardiac pulsatility. (**A**) Left: representative image of a pial arteriole taken using multiphoton microscopy. The vascular lumen was labelled with FITC-dextran (pseudocolor red) and Aβ deposits were labelled with methoxy-X04 (cyan). Scale bar = 50 μm. Right panel: trace showing diameter fluctuations over time of the same pial arteriole. (**B**) The diameter of pial arterioles did not differ between groups before treatment began (**C**) and there was no effect of treatment on vessel diameter over time. (**D**) Left panel: an example line scan path for the measurement of high frequency pulsations from the heartbeat. Scale bar = 25 μm. Right panel: example trace showing high frequency diameter measurements from a high-speed line scan. (**E**) The integral of the diameter traces generated from high-speed line scans did not differ between groups before treatment began (**F**) or throughout the treatment duration. Reported p-values from one way ANOVA (**B**), Kruskal Wallis test (**E**) or two-way repeated ANOVAs/mixed-effects analysis (**C, F**). No effect of sex was observed in HAE-4 or control IgG groups (PBS group lacked statistical power to test for sex). Each data point represents the animal average and solid lines (in **C** and **F**) represent average per treatment group. Males are represented by open symbols and females are represented by filled symbols. Error bars (in **B** and **E**) represent standard deviation. *N* = 6–10 animals per group (2–5 females / 4–5 males per group)

**Fig. 3:**
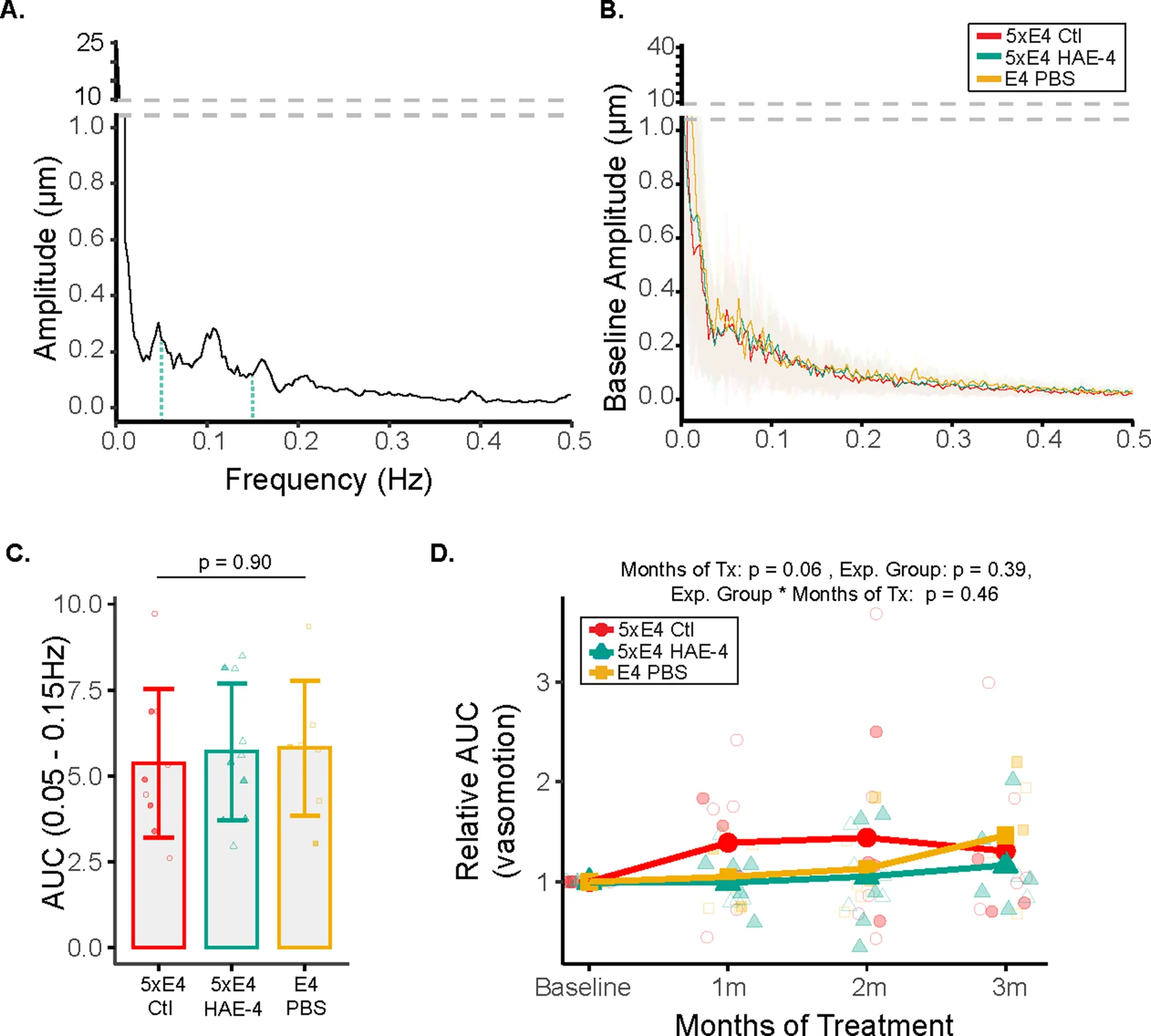
HAE-4 treatment does not alter low frequency vasomotion. (**A**) Example power spectrum showing the frequency of oscillations of diameter trace shown in Fig. 2a. Dashed lines show the frequencies (0.05–0.15 Hz) between which area under curve (AUC) values were calculated. (**B**) Average power spectra for each treatment group. (**C**) AUC values between 0.05–0.15 Hz did not differ between groups at baseline, before treatment began. (**D**) There was no effect of treatment on the relative AUC over time. Reported p-values from one way ANOVA (**C**) or two-way repeated ANOVA/mixed-effects analysis (**D**). No effect of sex was observed in HAE-4 or control IgG groups (PBS group lacked statistical power to test for sex). Each data point represents the animal average and solid lines (in **D**) represent average per treatment group. Males are represented by open symbols and females are represented by filled symbols. Shaded areas (in **B**) and error bars (in **C**) represent standard deviation. *N* = 6–10 animals per group (2–5 females / 4–5 males per group)

Previous work suggested that vascular reactivity was superior in a cross section of mice that received HAE-4, however it remains unclear if the function of a single vessel might improve or deteriorate when measured longitudinally. To determine if the effects observed acutely were present in a chronic setting, the same vessels were imaged monthly for the duration of treatment. We included an additional control group of mice with human APOE4, but without the 5XFAD mutations, which received PBS throughout the treatment duration (E4-PBS). We measured the diameter of pial arterioles before the initiation of treatment and subsequently during the treatment period **(**Fig. 2A**)**. In a mouse model of CAA, vessel ‘bloating’ has been observed in conjunction with CAA accumulation [18], ostensibly as a result of the loss of vascular tone due to the well documented smooth muscle cell damage that occurs alongside vascular Aβ accumulation [19]. We did not observe any differences in vascular diameter between treatment groups at baseline **(**Fig. 2B**)**, nor in relative diameter across the treatment duration **(**Fig. 2C**)**. Similarly, we have previously observed an increase in cardiac beat-to-beat pulsatility [18], presumably also due to the loss of vascular tone. We also did not see any difference across groups before the initiation of treatment **(**Fig. 2D-E**)** or throughout the treatment duration **(**Fig. 2F**)**. Finally, we have previously reported that the spontaneous low frequency (~ 0.1 Hz) fluctuations in vascular diameter, termed vasomotion, are a potentially important driving force for perivascular Aβ clearance [20] which deteriorates with CAA [18]. We transformed vascular diameter traces from the time domain to the frequency domain and measured the AUC between 0.05 and 0.15 Hz to get a measure of power at this low frequency vasomotion range **(**Fig. 3A-B**)**. We chose this frequency range to center around the ‘classic’ vasomotion frequency of 0.1 Hz [20]. We saw no difference in vasomotion power between groups before the initiation of treatment **(**Fig. 3C**)**, nor any effect of treatment on relative power throughout the treatment duration **(**Fig. 3D**)**.

### HAE-4 treatment did not alter stimulus-evoked responses in pial vessels over time

**Fig. 4:**
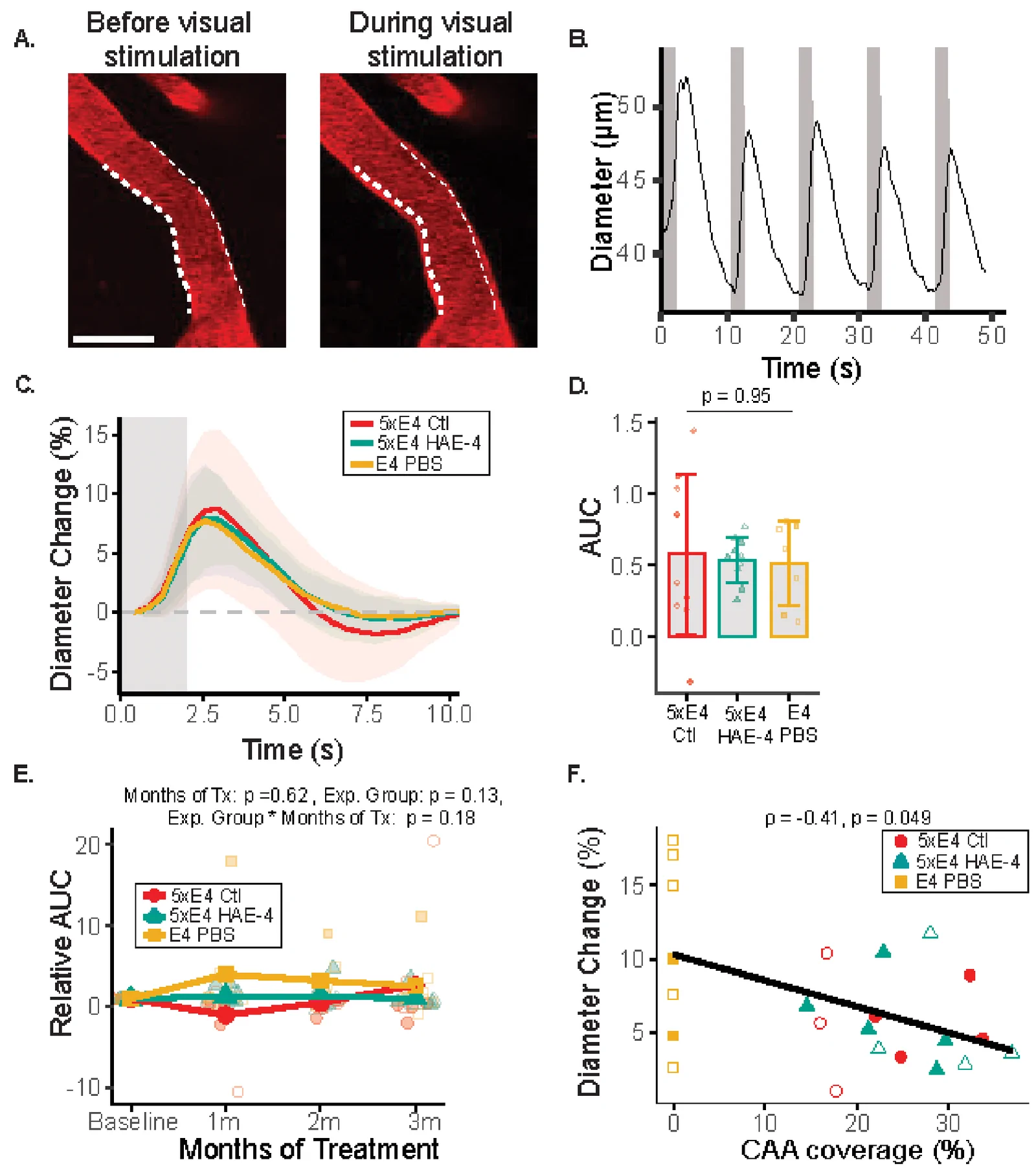
HAE-4 treatment does not alter visually evoked vascular responses. (**A**) Example pial arteriole in the visual cortex before (left) and during (right) visual stimulation. Scale bar = 50 μm. (**B**) Diameter trace of arteriole shown in **A**) during visual stimulation (shaded area). (**C**) Average visually evoked responses for each group, before the initiation of treatment, (**D**) did not differ. (**E**) There was no effect of treatment on relative area under curve (AUC) over time, but (**F**) responses did corelate negatively with the absolute CAA burden at the final imaging session. Reported p-values from one way ANOVA (**D**), two-way repeated ANOVA/mixed-effects analysis (**E**) or Spearman’s ρ (**F**). No effect of sex was observed in HAE-4 or control IgG groups (PBS group lacked statistical power to test for sex). Each data point represents the animal average and solid lines (in **C** and **E**) represent average per treatment group. Males are represented by open symbols and females are represented by filled symbols. Shaded areas (in **C**) and error bars (in **D**) represent standard deviation. *N* = 6–10 animals per group (2–5 females / 4–5 males per group)

Neuronal activation of the visual cortex, by the presentation of a flashing checkerboard, stimulates the release of vasoactive messengers and, in a healthy system, results in the relaxation of vascular mural cells and a vascular dilation **(**Fig. 4A-B**)**, termed the neurovascular coupling response [14]. In both mouse models of CAA [18, 20] and human individuals [21], this response eventually deteriorates with the accumulation of pathology and thus is a sensitive and translatable read-out of CAA-mediated vascular dysfunction. When we averaged the magnitude of vascular dilation in response to the visual stimuli (grey bars in Fig. 4B) across trials for each mouse, we did not observe any differences in vascular reactivity between groups prior to the initiation of treatment **(**Fig. 4C-D**)** and the relative magnitude remained unchanged throughout the treatment duration **(**Fig. 4E**)**. To validate the sensitivity of this measure to CAA burden across all treatment groups, we correlated the amplitude of vascular reactivity with the CAA coverage with the last imaging session. As expected, the amplitude of vascular reactivity was correlated with CAA burden in mice at the last imaging session **(**Fig. 4F**)**, indicating that this measure remained sensitive to vascular Aβ burden in this study. Therefore, the relatively unchanging vascular response to stimulation is commensurate with the observation that the CAA was not actively removed from these vessels. It is important to acknowledge that, without the inclusion of the E4 PBS group, this correlation is no longer significant (*r* = -0.16, *p* = 0.56).

Together, these data show largely unchanged vascular function with HAE-4 treatment, suggesting that the observed plaque removal did not result in any functional damage to the vasculature, nor did it improve vascular function beyond baseline, likely due to the absence of active CAA removal in the regions of the brain imaged in vivo.

### HAE-4 treated animals had less total Aβ than those treated with control IgG, without observed hemorrhagic events

**Fig. 5:**
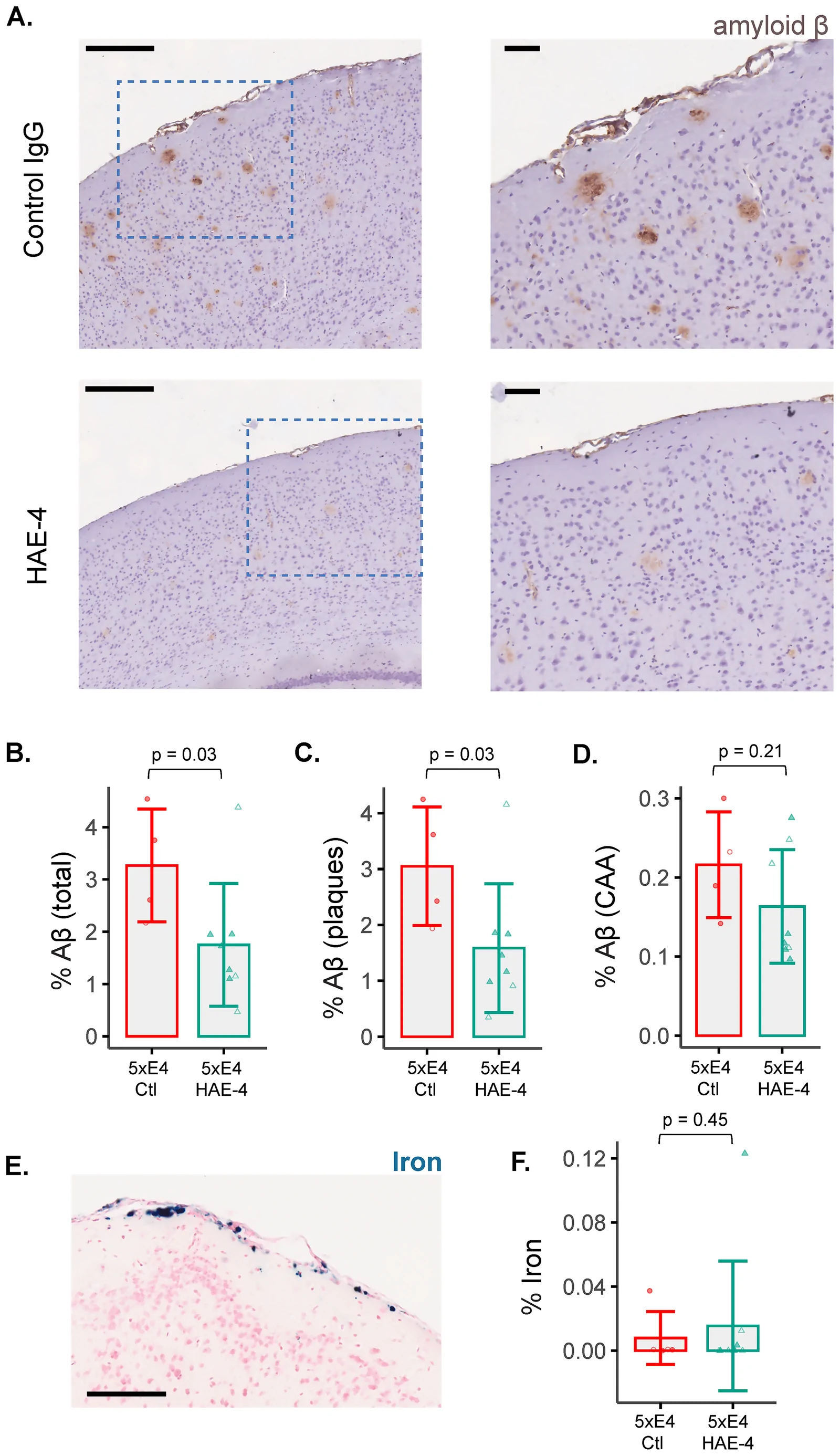
HAE-4 treatment does reduce overall levels of cortical Aβ. (**A**) Representative images of sections from the control IgG group (top panel) and HAE-4 group (bottom panel) that underwent immunohistochemistry against Aβ (clone 6 F/3D). Scale bar (left) = 200 μm, scale bar (right) = 50 μm. Right panel shows a higher magnification image of blue boxes from the left panel. (**B**) The percentage of Aβ positive cortical tissue was lower in HAE-4 treated mice, (**C**) an effect that was driven by lower plaque area. (**D**) Levels of CAA were not lower in HAE-4 treated mice. (**E**) Image of section with a hemorrhage that underwent Perls’ Prussian blue staining for iron deposits. Scale bar = 100 μm. (**F**) The percentage of iron positive tissue did not differ between groups. Each data point represents the animal average. Males are represented by open symbols and females are represented by filled symbols. Reported p-values from Mann-Whitney U tests and lacked statistical power to test for sex. Error bars (in **B, C, D**, and **F**) represent standard deviation. *N* = 4–8 animals per group (1–5 females / 2–5 males per group)

Following the completion of treatment, the brain was removed from the mice and stained for Aβ. As expected, significantly lower levels of total cortical Aβ were measured **(**Fig. 5A-B**)**. This reduction was driven by a reduction in parenchymal plaques **(**Fig. 5C**)** with a non-significant numerically lower mean in vascular Aβ deposits **(**Fig. 5D**)**. Staining with Perls’ Prussian blue revealed a low number of hemorrhagic lesions, without any effect of treatment group **(**Fig. 5E-F**)**. It is worth noting, however, that two large hemorrhages were observed **(**Fig. 5E**)**. As we saw one in each group they were unlikely related to drug treatment.

Taken together, post-mortem data indicate that, as previously documented, administration of HAE-4 immunotherapy resulted in lower overall levels of Aβ in the cortex, without concomitant hemorrhages.

### Aβ reduction in HAE-4 treated animals was associated with decreased Aβ deposition and immune cell activation in a separate cohort of mice

**Fig. 6:**
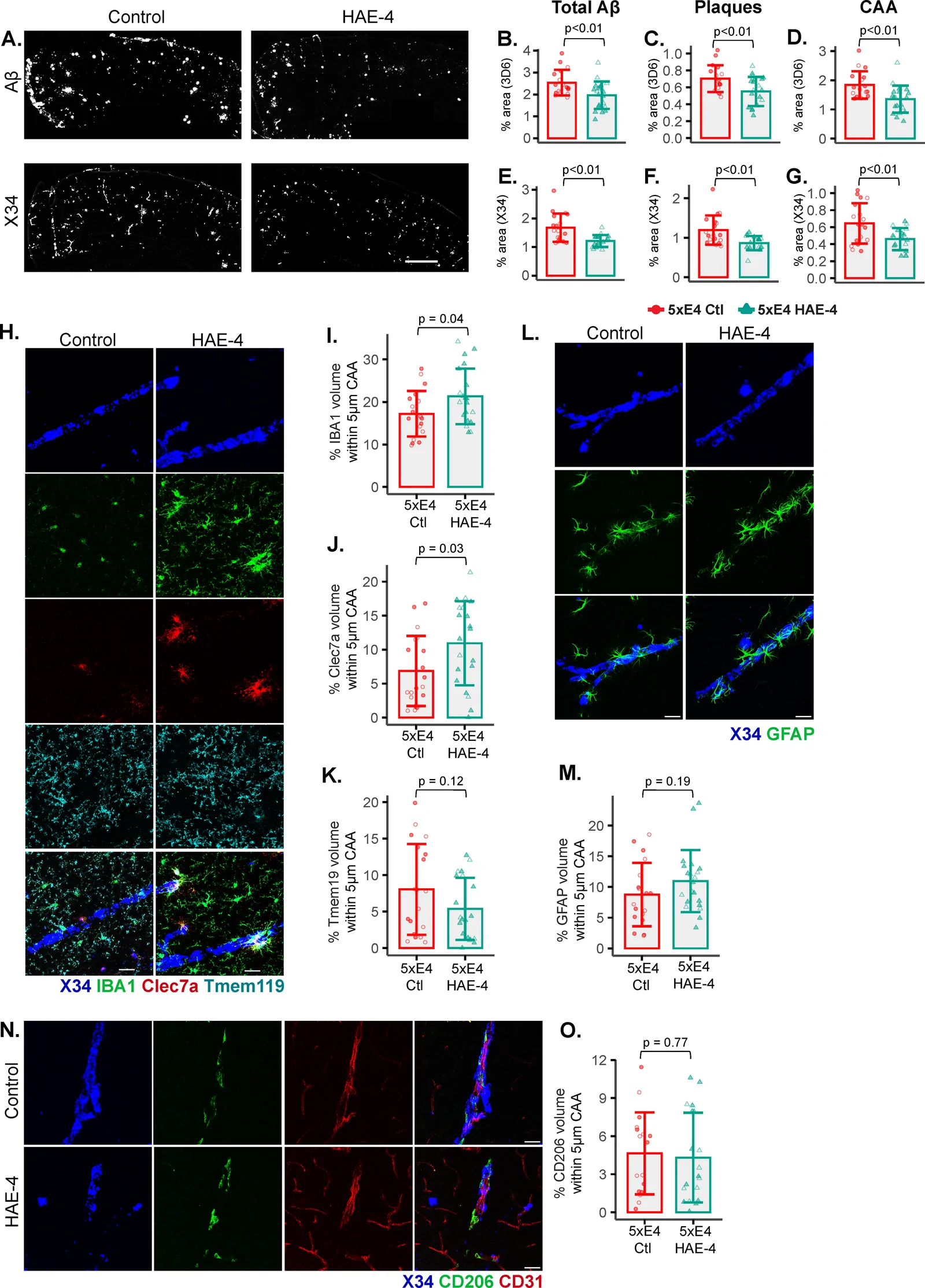
HAE-4 treatment in a separate cohort of mice reduces parenchymal and vascular Aβ and is associated with microglial activation. (**A**) Representative brain sections stained for total Aβ immunoreactivity (3D6) and X34 (fibrillar Aβ) from the control IgG group and HAE-4 group, Scale bar 500 μm. (**B**) The percent cortical area covered by total Aβ, (**C**) parenchymal plaques, and (**D**) levels of CAA were lower in HAE-4-treated mice. (**E**) The percent cortical area covered by total X34, (**F**) parenchymal plaques, and (**G**) CAA were lower in HAE-4-treated mice. (**H**) Representative confocal images of IBA1 (green), Clec7a (red), Tmem119 (cyan), co-stained with X34 (blue), in the cortex of mice treated with control IgG and HAE-4, scale bar 50 μm. (**I**) The percentage of Iba1 + voxels and (**J**) Clec7a+ voxels within 5 μm of X34 + CAA is higher in the HAE-4-treated mice. **K**) No significant change in the percentage area coverage of Tmem119 + voxels within 5 μm around X34 + CAA is noted. **L**) Representative confocal images of GFAP (green), co-stained with X34 (blue), CAA in the cortex of control IgG and HAE-4 treated mice, scale bar 50 μm. **M**) The percentage of GFAP+ voxels within 5 μm of X34 + CAA is unaltered between the control IgG group and HAE-4 group. **N**) Representative confocal images of CD206 (green), CD31 (red), co-stained with X34 (blue), CAA in the cortex of control IgG and HAE-4 treated mice, scale bar = 50 μm. **O**) The percentage of CD206 + voxels within 5 μm around X34 + CAA is unaltered between the control IgG group and HAE-4 group. Reported p-values from students t-tests. No effect of sex was observed in HAE-4 or control IgG groups. Each data point represents the animal average. Males are represented by open symbols and females are represented by filled symbols. Error bars represent standard deviation. *N* = 17–21 animals per group (9–14 females / 6–9 males per group)

In order to examine inflammatory responses to HAE-4 treatment in a larger cohort, we treated a new group of 5xE4 mice with moderate CAA who did not undergo cranial window surgery (see Supplementary Methods). Mice received weekly i.p. injections of either 50 mg/kg of HAE-4 anti-ApoE antibody or control IgG for 8 weeks starting at 8 months of age as was done previously (see Supplementary Methods for details) [10]. At the end of the treatment, mice were sacrificed, and brains were extracted following transcardial perfusion for histological analyses. As shown previously [10], the HAE-4 treatment group showed significantly lower levels of Aβ immunoreactive plaques as well as X34 + fibrillar plaques in the cortex **(**Fig. 6A, B, E**)** and hippocampus (Supplementary Fig. 2A-D). This effect was due to a reduction in both parenchymal plaques (Fig. 6C, F) and CAA (Fig. 6D, G). HAE-4 did not cause an increase in antibody-induced microhemorrhages (Supplementary Fig. 2G, H) or fibrinogen staining (Supplementary Fig. 2E, F) relative to control IgG. We further studied immune cell responses around the CAA by assessing reactive microglia (Fig. 6H), astrocytes (Fig. 6L), and perivascular border macrophages (Fig. 6N). Results showed a significant increase in the percentage area covered by Iba1+ (Fig. 6I) and Clec7a+ (Fig. 6J) reactive microglia in the 5 μm area around the CAA in HAE-4 treated mice compared to control IgG, while no change was observed in Tmem119 + percent area coverage (Fig. 6K). GFAP+ astrocytes (Fig. 6L, M) and CD206 + perivascular border macrophages (Fig. 6N, O) were present around the CAA but showed no difference between groups.

## Discussion

Previous work showed that 5XE4 mice had reduced levels of Aβ following two months of treatment with HAE-4, an anti-ApoE antibody, notably without concomitant hemorrhagic events. In addition, vascular reactivity was greater compared to control mice in acute experiments and inflammatory profiles were altered [10]. In this current study we set out to expand upon this work to longitudinally image the same pial blood vessels over time, in vivo, to determine if existing deposits of CAA could be removed with HAE-4 immunotherapy. Further, we sought to investigate if targeting non-lipidated ApoE4 that co-deposits with CAA would alter vascular function over time, either negatively or positively. In accordance with previous work, we found less Aβ by immunostaining in post-mortem tissue accompanied by an increase in perivascular reactive microglia, without any evidence of HAE-4 treatment causing hemorrhage. By conducting chronic longitudinal in vivo imaging, we found that a subset of the smallest fibrillar plaques reduced in size over time with HAE-4 treatment, but that fibrillar CAA was not actively removed from vessels in the brain region imaged with in vivo multiphoton microscopy under a cranial window. In line with these observations, we did not see any improvement or deterioration in vascular function in those same vessels.

At first glance, removal of vascular Aβ deposits with anti-Aβ immunotherapy appears to be a conceptually reasonable therapeutic approach, but despite recent advances in the AD field, clinicians have advised against the use of anti-Aβ antibodies for the treatment of CAA [5]. This is largely because of CAA’s associated increased risk for the development of ARIA, common adverse events seen with anti-Aβ immunotherapy in AD patients. ARIA-H (hemorrhage) and ARIA-E (edema) have been reported in a significant number of participants receiving anti-Aβ immunotherapy, are dose dependent and although largely resolve upon cessation of treatment, can have serious consequences [1, 4]. ARIA-H is characterized by hemorrhagic imaging markers such as microbleeds and cortical superficial siderosis, and ARIA-E by edematous effusions [1]. Data show that the presence of cortical microbleeds at baseline, indicative of underlying CAA, elevates the risk of ARIA, as does the possession of an APOE4 allele [1, 6]. Therefore, administration of certain anti-Aβ immunotherapy potentiates the bleeding risk of an already vulnerable population and an alternative is urgently needed.

Although the mechanism of ARIA remains unclear, accumulating evidence suggests a role for perivascular inflammation, where administered antibodies target CAA on fragile vessels and induce a local immune response, resulting in vessel wall breakdown and subsequent leakage [1, 22]. In patients with sporadic non-inflammatory CAA, the occurrence of vascular remodeling in severely affected blood vessels has also been associated with increases in perivascular inflammation which is postulated to contribute to subsequent vessel wall breakdown [23]. Indeed, the previous study investigating the effect of HAE-4 in 5xE4 mice [10] showed that there was an acutely potentiated inflammatory response alongside an increased parenchymal response around Aβ plaques by microglia. This increased parenchymal response decreased over 8 weeks. The profile was different to that of aducanumab-treated mice where there was a more prolonged immune response dominated by astrocytes, which correlated with the number of microbleeds on post-mortem examination [10]. Alongside existing work, our data showing reduced total Aβ post-mortem and the shrinking of plaques in vivo without evidence for ARIA-like events, raises the possibility that HAE-4 immunotherapy is associated with less ARIA risk than anti-Aβ immunotherapy in mice. The question of the optimal timing of antibody administration remains an open one. Since the current in vivo data suggest that CAA was not actively removed from pial surface vessels, and only the smallest of intraparenchymal plaques reduced in size, earlier administration is likely to be more successful to prevent further Aβ build-up. Notably, since the reduction in total Aβ deposits as detected by immunostaining was greater than that measured in vivo using methoxy-X04, it implies that HAE-4 was more effective at removing aggregated but non-fibrillar Aβ in this study, which could be quantified by directly measuring levels of different Aβ species in future work. The completion of further investigations in both younger and older animals would aid in pinpointing the ideal window for intervention.

Aside from the hemorrhagic consequences, the effect of enhanced perivascular inflammation on vascular function remains a subject of concern. In both sporadic and Dutch-type CAA, visually evoked vascular reactivity measured with BOLD fMRI declines early in the disease trajectory, before the occurrence of hemorrhagic lesions [21, 24]. Vascular reactivity is therefore considered a highly informative outcome marker in clinical trials for patients with CAA and is thus an important readout from animal studies aiming to develop therapeutics. Paradoxically, a clinical trial in patients with CAA who received the anti-Aβ antibody ponezumab, showed a trend towards reduced BOLD responses to visual stimulation [25]. Although the reasons for this observation are unconfirmed, one possibility is the mobilization of Aβ from plaques towards the vasculature, thereby increasing CAA burden and reducing vascular function. We observed no evidence for this redistribution of Aβ in our study. Alternatively, it is tempting to speculate that there may have been recruitment of immune cells towards the vasculature, and a pro-inflammatory milieu that led to a decline in vascular function. In our study, even without active CAA removal from the vasculature, an antibody-mediated increase in perivascular microglia could have had detrimental effects on vascular function. However, our data showed that there was no alteration to the measured vascular responses upon treatment with HAE-4, either positively or negatively, but rather they remained relatively stable across the treatment duration. It remains to be seen if vascular function can be restored once it has begun to decline. Studies in older animals with more advanced pathology should be used in the future to answer this question as our 10-month-old 5XE4 animals did not display a baseline deficit in vascular function when compared to APOE4 mice without Aβ pathology.

Of note, we did not observe any alterations to vascular reactivity following the administration of HAE-4 under the cranial window, but previous work reported better outcomes across several measures of vascular function [10]. There are several methodological and context-dependent differences worth pointing out here, that could potentially underly this apparent discrepancy. First, the previous study [10] assessed vessels at a single time point, and no information was collected regarding baseline vessel properties. It can therefore not be established whether active CAA clearance from the blood vessels occurred and whether function was restored versus whether further deterioration was prevented. This was the main reason we set out to perform the current study, utilizing longitudinal in vivo multiphoton imaging. To our knowledge, there has never been strong evidence from longitudinal in vivo imaging studies for the removal of existing CAA from blood vessels in transgenic mice. It is therefore not completely surprising that the current study also did not reveal any ‘active’ CAA clearance – and corresponding vascular functional recovery – over time. Second, the previous study [10] compared vessel diameter changes in response to hypercapnia, SNAP, and Ach which primarily assess intrinsic vasodilatory capacity by directly stimulating vascular smooth muscle and endothelial pathways [10]. The current study compared vessel diameter changes in response to visual stimulation, which relies on neurovascular coupling and requires coordinated signaling between neurons, astrocytes, endothelial cells, and vascular smooth muscle cells [26]. Thus, the current readout reflects integrated neurovascular unit function rather than isolated vessel reactivity, which might respond differently to HAE-4 treatment. Third, the surgical preparation was also different. In the previous study [10] a terminal open craniotomy was performed to assess vascular function during a single acute imaging session. In the current study, a chronic cranial window was implanted, and imaging was performed longitudinally over 12 weeks. We cannot entirely rule out the impact of the cranial window implant itself on our observed results, as it is an invasive procedure. Having said that, previous literature suggests that implantation of a cranial window does not substantially influence target engagement of peripherally administered antibodies [27]. This was also demonstrated by our observation of shrinkage of the smallest plaques over time in our in vivo data. The two primary pathways hypothesized to underly the previously reported reductions in Aβ following HAE-4 treatment are through elimination by microglia (removal of Aβ plaques by HAE-4 requires myeloid cells as mutating the Fcγ receptor of the HAE-4 antibody was found to block the effects of HAE-4 on parenchymal Aβ reduction [11]) or perivascular clearance [10]. There is extensive evidence for preserved perivascular movement of solutes under cranial windows (e.g [20, 28, 29]). Previous work has also shown only a modest increase in the Iba1 positive inflammatory profile measured under chronic (neocortical) cranial windows when compared to non-surgical hemispheres [30], suggesting minimal impact of the surgery on microglial dynamics when allowed sufficient time to recover. Having said that, future work is needed to fully interrogate the potential impact of surgical preparation on tracking in vivo CAA clearance, through both mechanisms, and considering recent data supporting the notion that peripherally administered antibodies enter the brain first along superficial CSF pathways [31]. Fourth, experimental evidence shows that the neurovasculature in mice with Aβ pathology is particularly sensitive to invasive procedures, inducing cortical spreading depressions and altered haemodynamics [32]. In the present study, mice were allowed to recover for one month before the commencement of the initial imaging session, were imaged awake and had a sealed cranial window. Conversely, the experiments reporting vascular dysfunction in mice with higher global levels of Aβ were conducted acutely on the same day as surgery, with an open window preparation and under anesthesia [10]. It is possible that the prior observations reflect the inability of the brain to maintain physiological responses during acute stress when more heavily burdened with Aβ, as has previously been reported [32]. Fifth, a major limitation of the current study is that we only followed arterioles already with existing CAA at the start of the experiment, rather than also recording from arterioles that did not have CAA prior to treatment. A more network-wide approach would have helped with understanding if HAE-4 is helping to prevent aggregation on vessels that were preserved at baseline.

Moreover, whereas we observed a striking reduction of Aβ in post-mortem tissue in mice treated with HAE-4, yet only a very modest shrinkage of the smallest plaques when the same pathology was repeatedly imaged in vivo. There are several considerations that could account for this apparent discordance. In particular, our in vivo data were recorded from a relatively small and superficial FOV when compared to the (cortical hemisphere) post-mortem histopathology. Consequently, the in vivo data show only a small fraction of Aβ pathology that are biased towards the pial surface vessels and the first layer(s) of cortical parenchyma. Additionally, our research question of whether or not HAE-4 immunotherapy can actively remove CAA that is already deposited, necessitated the imaging of vessels that already had CAA before the commencement of treatment. Therefore, our in vivo data do not answer the question of whether or not HAE-4 immunotherapy might slow or prevent the accumulation of new Aβ deposits in the vasculature and does not give a macroscopic view of pathology. Future work monitoring the effect of immunotherapy on the blood vessels that do not have CAA at the beginning of treatment would help to unravel the mechanism underlying our observed results.

Although we similarly saw a reduction in the degree of Aβ post-mortem with HAE-4 treatment, this was driven by a lower plaque burden rather than there being less CAA, in contrast to what was observed previously and in a separate cohort of mice included here (Fig. 6) [10]. There are several possible explanations for this. First, pial CAA burden in this mouse model is high relative to other vessel types and due to our window preparations, it is difficult to avoid damaging the leptomeninges when removing the brain post-mortem, thereby losing some pial vasculature. As a result, it is likely that the total number of pial arterioles is lower in our sections and thus any reductions in CAA might have been underestimated. Second, in this study, post-mortem brains were processed, paraffin embedded, and cut thinly, whereas samples were previously fixed and then cut using a freezing microtome in thicker Sect [10]. Third, in our post-mortem study of surgically prepared mice, we have small sample sizes due to unforeseen technical issues (e.g. damage during processing) making this particular experiment underpowered. Fourth, this study utilized mice on a different background to that used previously [10]. In order to reconcile these differences and ensure the reproducibility of previous findings, we treated a separate, larger, sufficiently powered – albeit slightly younger – cohort of mice with the same background that were not surgically prepared. In this cohort we found a reduction in CAA as well as plaques, confirming previous findings [10].

Future work should focus on unravelling the mechanism of action of HAE-4 immunotherapy. HAE-4 targets the non-lipidated subspecies of ApoE4, without affecting total ApoE levels in the brain [10, 11]. As mentioned, for parenchymal Aβ plaque removal, there is direct evidence that the prevention and removal of Aβ plaques by HAE-4 requires myeloid cells (likely microglia) as introduction of a mutation in the Fcγ receptor of the HAE-4 antibody blocks the effects of HAE-4 on parenchymal Aβ reduction [11]. If non-lipidated ApoE4 acts as a seed for Aβ deposition or aggregation, then the removal of this species with HAE-4 immunotherapy could help to prevent further accumulation. Alternatively, previous work demonstrated an upregulation in microglial associated genes as well as the recruitment of microglia around Aβ deposits. We reproduced this finding in this current study and expanded these data further to examine the effect of HAE-4 on perivascular macrophages and BBB leakage, neither of which were altered by treatment. Together, these data point towards enhancement of active cellular uptake as a potential underlying mechanism [10]. Further work is also required to better understand the pathophysiology underlying ARIA-H and with the use of MRI, ARIA-E. This will help with the development of safer therapeutics or adjunct therapies to be administered in combination with drugs targeting Aβ. Additional translational insights would be gained by examining the effect of HAE-4 administration on cognitive outcomes in models that display behavioral deficits and should be considered in future studies.

Finally, CAA is currently diagnosed clinically using MRI-visible manifestations such as microbleeds and enlarged perivascular spaces [33] which represent more advanced disease stages. Both our work and that done previously [10] studied mice with moderate levels of CAA to better recapitulate what might be observed in the human brain at an early detectable disease stage. To understand the potential of HAE-4 immunotherapy in preventing CAA or slowing down CAA disease progression, future studies should initiate treatment at a younger age, likely before the deposition of vascular Aβ.

## Conclusions

Together, these data support previous findings that HAE-4 can effectively reduce deposited Aβ but did not convincingly demonstrate active removal of existing CAA from already affected vessels in vivo. HAE-4 immunotherapy could be a safe alternative to anti-Aβ immunotherapy as Aβ load in treated mice was reduced without observed hemorrhages or decline in vascular function. However, because we found no evidence for active fibrillar CAA removal, the timing of treatment initiation is likely to be critical as it may be more useful for slowing CAA disease progression, rather than reversing any existing pathology or ongoing disease process. Future work will be necessary for understanding the full therapeutic potential of HAE-4 immunotherapy for CAA and AD.

## Supplementary Information

Below is the link to the electronic supplementary material.

Supplementary Material 1: Representative images of in vivo plaques from three different mice at baseline and following 3 months of treatment with HAE-4 or control IgG. Some plaques shrunk and some remained stable throughout the treatment duration. Scale bar = 200 μm.

Supplementary Material 2: (A) Representative hippocampal brain sections stained for X34 (fibrillar Aβ) from the control IgG group and HAE-4 group, Scale bar 500 μm. (B) The percent cortical area covered by total X34, (C) parenchymal plaques, and (D) levels of CAA were lower in HAE-4-treated mice compared to IgG treated mice. (E) Representative brain sections stained for fibrinogen from control IgG group and HAE-4 group, Scale bar = 50 μm. (F) The percentage of fibrinogen+ voxels is unaltered between the control IgG group and HAE-4 group. (G) The percentage of Prussian blue+ voxels per 10mm^2^ is unaltered between the control IgG group and HAE-4 group as well as those co-localized with Aβ (H). Reported p-values from students t-tests. No effect of sex was observed in HAE-4 or control IgG groups. Each data point represents the animal average. Males are represented by open symbols and females are represented by filled symbols. Error bars represent standard deviation. N = 14–18 animals per group (8–12 females / 5–8 males per group).

Supplementary Material 3

## Data Availability

The datasets used and/or analysed during the current study are available from the corresponding author on reasonable request.

## Abbreviations

Aβ: Amyloid-β
APOE: Apolipoprotein E
ARIA: Amyloid-related imaging abnormalities
ARIA-E: ARIA -edema
ARIA-H: ARIA -hemorrhage
CAA: Cerebral amyloid angiopathy
FOV: Field of view
HAE-4: Anti-ApoE4 immunotherapy

## References

[1] Greenberg SM, Bacskai BJ, Hernandez-Guillamon M, Pruzin J, Sperling R, van Veluw SJ. Cerebral amyloid angiopathy and Alzheimer disease — one peptide, two pathways. Nat Rev Neurol. 2020;16:30–42. 31827267 10.1038/s41582-019-0281-2 PMC7268202

[2] Jäkel L, De Kort AM, Klijn CJM, Schreuder FHBM, Verbeek MM. Prevalence of cerebral amyloid angiopathy: A systematic review and meta-analysis. Alzheimer’s Dement. 2022;18:10–28. 34057813 10.1002/alz.12366 PMC9290643

[3] Budd Haeberlein S, Aisen PS, Barkhof F, Chalkias S, Chen T, Cohen S, et al. Two Randomized Phase 3 Studies of Aducanumab in Early Alzheimer’s Disease. J Prev Alzheimers Dis. 2022;9:197–210. 35542991 10.14283/jpad.2022.30

[4] Greenberg SM, Bax F, van Veluw SJ. Amyloid-related imaging abnormalities: manifestations, metrics and mechanisms. Nat Rev Neurol. 2025;21:193–203. 39794509 10.1038/s41582-024-01053-8

[5] Greenberg SM, Cordonnier C, Schneider JA, Smith EE, van Buchem MA, van Veluw SJ, et al. Off-label use of aducanumab for cerebral amyloid angiopathy. Lancet Neurol. 2021;20:596–7. 34237272 10.1016/S1474-4422(21)00213-1

[6] Salloway S, Chalkias S, Barkhof F, Burkett P, Barakos J, Purcell D, et al. Amyloid-Related Imaging Abnormalities in 2 Phase 3 Studies Evaluating Aducanumab in Patients With Early Alzheimer Disease. JAMA Neurol. 2022;79:13–21. 34807243 10.1001/jamaneurol.2021.4161 PMC8609465

[7] Greenberg SM, Briggs ME, Hyman BT, Kokoris GJ, Takis C, Kanter DS, et al. Apolipoprotein E ε4 Is Associated With the Presence and Earlier Onset of Hemorrhage in Cerebral Amyloid Angiopathy. Stroke. 1996;27:1333–7. 8711797 10.1161/01.str.27.8.1333

[8] Fryer JD, Simmons K, Parsadanian M, Bales KR, Paul SM, Sullivan PM, et al. Human Apolipoprotein E4 Alters the Amyloid-β 40:42 Ratio and Promotes the Formation of Cerebral Amyloid Angiopathy in an Amyloid Precursor Protein Transgenic Model. J Neurosci. 2005;25:2803–10. 15772340 10.1523/JNEUROSCI.5170-04.2005 PMC6725147

[9] Nelson PT, Pious NM, Jicha GA, Wilcock DM, Fardo DW, Estus S, et al. APOE-ε2 and APOE-ε4 correlate with increased amyloid accumulation in cerebral vasculature. J Neuropathol Exp Neurol. 2013;72:708–15. 23771217 10.1097/NEN.0b013e31829a25b9 PMC3715146

[10] Xiong M, Jiang H, Serrano JR, Gonzales ER, Wang C, Gratuze M, et al. APOE immunotherapy reduces cerebral amyloid angiopathy and amyloid plaques while improving cerebrovascular function. Sci Transl Med. 2021;13:eabd7522. 33597265 10.1126/scitranslmed.abd7522 PMC8128342

[11] Liao F, Li A, Xiong M, Bien-Ly N, Jiang H, Zhang Y, et al. Targeting of nonlipidated, aggregated apoE with antibodies inhibits amyloid accumulation. J Clin Invest. 2018;128:2144–55. 29600961 10.1172/JCI96429 PMC5919821

[12] Huynh TPV, Wang C, Tran AC, Tabor GT, Mahan TE, Francis CM et al. Lack of hepatic apoE does not influence early Aβ deposition: observations from a new APOE knock-in model. Mol Neurodegener. 2019;14. 10.1186/s13024-019-0337-1 PMC6796484 31623648

[13] Saadi F, Katritch IV, Parhizkar S, Lugo B, Cho Y, Ulrich JD, et al. Maternal Inheritance Suppresses CAA and Amyloid Plaques in 5xFAD (Tg7031)/APOE4 Mice. Neuron. 2025;113:2883. 40967182 10.1016/j.neuron.2025.08.008 PMC12453590

[14] Munting LP, Bonnar O, Kozberg MG, Auger CA, Hirschler L, Hou SS, et al. Spontaneous vasomotion propagates along pial arterioles in the awake mouse brain like stimulus-evoked vascular reactivity. J Cereb Blood Flow Metab. 2023;43:1752–63. 36655606 10.1177/0271678X231152550 PMC10581232

[15] Schindelin J, Arganda-Carreras I, Frise E, Kaynig V, Longair M, Pietzsch T et al. Fiji: an open-source platform for biological-image analysis. Nature Methods. 2012;9:676–82. 10.1038/nmeth.2019 PMC3855844 22743772

[16] Bankhead P, Loughrey MB, Fernández JA, Dombrowski Y, McArt DG, Dunne PD, et al. QuPath: Open source software for digital pathology image analysis. Sci Rep. 2017;2017 7:1. 10.1038/s41598-017-17204-5 PMC5715110 29203879

[17] R Core Team. R: A language and environment for statistical computing. R Foundation for Statistical Computing; 2022.

[18] Kozberg MG, Munting LP, Hanlin LH, Auger CA, van den Berg ML, Denis, de Senneville B et al. Vasomotion loss precedes impaired cerebrovascular reactivity and microbleeds in cerebral amyloid angiopathy. Brain Commun. 2025;7:fcaf186. 10.1093/braincomms/fcaf186 PMC12096159 40406166

[19] Koemans EA, Chhatwal JP, van Veluw SJ, van Etten ES, van Osch MJP, van Walderveen MAA, et al. Progression of cerebral amyloid angiopathy: a pathophysiological framework. Lancet Neurol. 2023;22:632–42. 37236210 10.1016/S1474-4422(23)00114-X

[20] van Veluw SJ, Hou SS, Calvo-Rodriguez M, Arbel-Ornath M, Snyder AC, Frosch MP, et al. Vasomotion as a Driving Force for Paravascular Clearance in the Awake Mouse Brain. Neuron. 2020;105:549–61. 31810839 10.1016/j.neuron.2019.10.033 PMC7028316

[21] van Opstal AM, van Rooden S, van Harten T, Ghariq E, Labadie G, Fotiadis P, et al. Cerebrovascular function in presymptomatic and symptomatic individuals with hereditary cerebral amyloid angiopathy: a case-control study. Lancet Neurol. 2017;16:115–22. 27989553 10.1016/S1474-4422(16)30346-5 PMC5505183

[22] Taylor X, Noristani HN, Fitzgerald GJ, Oluoch H, Babb N, McGathey T, et al. Amyloid-β (Aβ) immunotherapy induced microhemorrhages are linked to vascular inflammation and cerebrovascular damage in a mouse model of Alzheimer’s disease. Mol Neurodegener. 2024;19:77. 39434125 10.1186/s13024-024-00758-0 PMC11494988

[23] Kozberg MG, Yi I, Freeze WM, Auger CA, Scherlek AA, Greenberg SM, et al. Blood-brain barrier leakage and perivascular inflammation in cerebral amyloid angiopathy. Brain Commun. 2022;4:fcac245. 36267331 10.1093/braincomms/fcac245 PMC9576155

[24] Switzer AR, McCreary C, Batool S, Stafford RB, Frayne R, Goodyear BG, et al. Longitudinal decrease in blood oxygenation level dependent response in cerebral amyloid angiopathy. Neuroimage Clin. 2016;11:461–7. 27104140 10.1016/j.nicl.2016.02.020 PMC4827726

[25] Leurent C, Goodman JA, Zhang Y, He P, Polimeni JR, Gurol ME, et al. Immunotherapy with ponezumab for probable cerebral amyloid angiopathy. Ann Clin Transl Neurol. 2019;6:795–806. 31020004 10.1002/acn3.761 PMC6469253

[26] Hall CN, Howarth C, Kurth-Nelson Z, Mishra A. Interpreting bold: Towards a dialogue between cognitive and cellular neuroscience. Philosophical Trans Royal Soc B: Biol Sci. 2016;371:1–12. 10.1098/rstb.2015.0348 PMC5003850 27574302

[27] Kastanenka KV, Bussiere T, Shakerdge N, Qian F, Weinreb PH, Rhodes K, et al. Immunotherapy with Aducanumab Restores Calcium Homeostasis in Tg2576 Mice. J Neurosci. 2016;36:12549–58. 27810931 10.1523/JNEUROSCI.2080-16.2016 PMC5157102

[28] Iliff JJ, Wang M, Liao Y, Plogg BA, Peng W, Gundersen GA, et al. A paravascular pathway facilitates CSF flow through the brain parenchyma and the clearance of interstitial solutes, including amyloid β. Sci Transl Med. 2012;4:147ra111. 22896675 10.1126/scitranslmed.3003748 PMC3551275

[29] Holstein-Rønsbo S, Gan Y, Giannetto MJ, Kaag Rasmussen M, Sigurdsson B, Ralf M, Beinlich F, et al. Glymphatic influx and clearance are accelerated by neurovascular coupling Check for updates. Nat Neurosci |. 2023;26:1042–53. 37264158 10.1038/s41593-023-01327-2 PMC10500159

[30] Shaw K, Bell L, Boyd K, Grijseels DM, Clarke D, Bonnar O, et al. Neurovascular coupling and oxygenation are decreased in hippocampus compared to neocortex because of microvascular differences. Nat Commun. 2021;12:3190. 34045465 10.1038/s41467-021-23508-y PMC8160329

[31] Pizzo ME, Plowey ED, Khoury N, Kwan W, Abettan J, DeVos SL, et al. Transferrin receptor-targeted anti-amyloid antibody enhances brain delivery and mitigates ARIA. Science. 2025;389:eads3204. 40773560 10.1126/science.ads3204

[32] Shabir O, Pendry B, Lee L, Eyre B, Sharp PS, Rebollar MA, et al. Assessment of neurovascular coupling and cortical spreading depression in mixed mouse models of atherosclerosis and Alzheimer’s disease. Elife. 2022;11:e68242. 35014950 10.7554/eLife.68242 PMC8752088

[33] Charidimou A, Boulouis G, Frosch MP, Baron JC, Pasi M, Albucher JF, et al. The Boston Criteria v2.0 for cerebral amyloid angiopathy: A multicentre MRI-neuropathology diagnostic accuracy study. Lancet Neurol. 2022;21:714. 35841910 10.1016/S1474-4422(22)00208-3 PMC9389452

