# Supplementary material for: Longitudinal multiphoton imaging of cerebral amyloid angiopathy in response to anti-ApoE4 immunotherapy in mice
Source: Mol Neurodegener. 2026 Jun 11;21:49. doi: 10.1186/s13024-026-00957-x (PMC13488262; doi:10.1186/s13024-026-00957-x)
Supplement: Supplementary file 1 [file 13024_2026_957_MOESM3_ESM.docx]

**Supplementary methods**

In order to increase statistical power and to better recapitulate previous work, a second cohort of naive 5xE4 mice was treated with 50mg/kg of HAE-4 or control IgG for 8 weeks from 8-10 months.

Mice were sacrificed, transcardially perfused with ice-cold PBS containing 0.3% heparin and brains were extracted and later cut into 30μm coronal sections on a freezing microtome. Sections were permeabilized using 0.25% TBS-X for 20 minutes before incubation with X-34 (1:5000, Sigma Cat#SML1954). Samples were blocked using 3% goat serum in 0.25% TBS-X for 30 minutes before incubation with the primary antibody for two nights at 4°C unless stated otherwise below. Primary antibodies used were: 3D6 (1:3000, Gift from Ron Demattos, Eli Lilly), Iba1 (1:500, Novus Biologicals, Cat# NB100-1028, Goat), Clec7a (1:50, InvivioGen, Cat# mabg-mdect, Rat), Tmem119 (1:500, Cell Signaling Technology, Cat# 90840, Rabbit), GFAP (1:500, Invitrogen, Cat# 53-9892-82, Mouse; incubated for 2 hours at room temperature), CD206 (1:500, BIO-RAD, Cat# MCA2235, Rat), Fibrinogen (1:500, Abcam, Cat# ab34269, Rabbit), and CD31 (1:250, Millipore Sigma, Cat# MAB1398Z, Armenian Hamster). Sections were incubated with the secondary antibody diluted in blocking buffer (donkey anti-rabbit IgG (H + L)-AlexaFluor647, 1:500; donkey anti-rat IgG (H + L)-AlexaFluor488, 1:500; donkey anti-goat IgG (H + L)-AlexaFluor594, 1:500; Goat anti-rat IgG (H + L)-AlexaFluor488, 1:500; Goat anti-Armenian hamster IgG (H + L)-AlexaFluor594, 1:250; Alexa Fluor™ 647 streptavidin, 1:1000; Invitrogen) for two hours at room temperature before being mounted and counterstained with True black and cover slipped with Prolong gold.

Slides were scanned Leica Stellaris 5 confocal microscope and Leica Application Suite X software (4.2.1.23810) and analyzed using Imaris software. For Aβ quantification, two sections contributed to each animal average. For percentage area within 5μm of CAA, two regions of interest per section were quantified across two sections. For Prussian blue staining for iron, 12-13 equally spaced sections were averaged per mouse.
